# Molar Incisor Hypomineralization, Prevalence, and Etiology

**DOI:** 10.1155/2014/234508

**Published:** 2014-05-08

**Authors:** Sulaiman Mohammed Allazzam, Sumer Madani Alaki, Omar Abdel Sadek El Meligy

**Affiliations:** ^1^Pediatric Dentistry Department, Faculty of Dentistry, King Abdulaziz University, P.O. Box 80209, Jeddah 21589, Saudi Arabia; ^2^Security Forces Dental Center, P.O. Box 1666, Alrass 51921, Saudi Arabia; ^3^Pediatric Dentistry & Dental Public Health Department, Faculty of Dentistry, Alexandria University, Alexandria 21521, Egypt

## Abstract

*Aim*. To evaluate the prevalence and possible etiological factors associated with molar incisor hypomineralization (MIH) among a group of children in Jeddah, Saudi Arabia. *Methods*. A group of 8-12-year-old children were recruited (n = 267) from the Pediatric Dental Clinics at the Faculty of Dentistry, King Abdulaziz University. Children had at least one first permanent molar (FPM), erupted or partially erupted. Demographic information, children's medical history, and pregnancy-related data were obtained. The crowns of the FPM and permanent incisors were examined for demarcated opacities, posteruptive breakdown (PEB), atypical restorations, and extracted FPMs. Children were considered to have MIH if one or more FPM with or without involvement of incisors met the diagnostic criteria. *Results*. MIH showed a prevalence of 8.6%. Demarcated opacities were the most common form. Maxillary central incisors were more affected than mandibular (*P* = 0.01). The condition was more prevalent in children with history of illnesses during the first four years of life including tonsillitis (*P* = 0.001), adenoiditis (*P* = 0.001), asthma (*P* = 0.001), fever (*P* = 0.014), and antibiotics intake (*P* = 0.001). *Conclusions*. The prevalence of MIH is significantly associated with childhood illnesses during the first four years of life including asthma, adenoid infections, tonsillitis, fever, and antibiotics intake.

## 1. Introduction

The term molar incisor hypomineralization (MIH) was introduced in 2001 to describe the clinical appearance of enamel hypomineralization of systemic origin affecting one or more first permanent molars (FPMs) that are associated frequently with affected incisors [1]. The condition is also known as nonfluoride enamel opacities, internal enamel hypoplasia, nonendemic mottling of enamel, opaque spots, idiopathic enamel opacities, enamel opacities, and idiopathic enamel hypomineralization [2]. A wide variation in the reported prevalence of MIH exists with rates varying from 3.6% to 37.5% [2, 3].

Clinically, the hypomineralized enamel can be soft, porous, or resembling discolored chalk or old Dutch cheese. The enamel defects can vary in color from white to yellow or brown, but they always show a sharp demarcation between the affected and sound enamels. The porous, brittle enamel can easily chip off under masticatory forces. Occasionally, loss of enamel can occur so rapidly after eruption that it seems as if the enamel was not formed initially and giving a picture resembling hypoplasia. The latter, however, has smooth margins to the surrounding enamel, whilst in MIH the borders appear to be irregular [4].

Molar incisor hypomineralization can sometimes present with opacities in the upper and lower incisors. The risk of defects to the upper incisors appears to increase with increasing number of affected FPMs. The defects of incisors are usually without loss of enamel substance and are generally less serious than those seen in molars due to absence of chewing forces [5]. The second permanent molars and bicuspids are very seldom impaired by these enamel defects [6]. According to Weerheijm et al., the second primary molars, second permanent molars, and the tips of the permanent canines can also show enamel defects occasionally [7].

Defective molar teeth are more susceptible to plaque accumulation and dental caries and may therefore have a greater need for dental treatment [8, 9]. Because the prismatic morphology in the porous enamel is altered, bonding to enamel becomes difficult leading to frequent loss of fillings and repeated treatment [10, 11]. Children with affected molars generally receive more dental treatments than those without and a significant number of retreated teeth eventually require extraction [12, 13].

The treatment of teeth with MIH can be painful due to difficulties in anaesthetizing, most likely due to subclinical inflammation of the pulpal cells caused by the porosity of the enamel. Because of the difficulties in achieving adequate anesthesia and frequent treatments, children with hypomineralized first molars may show difficult behavior and dental fear and anxiety [12].

The aim of the present study was to evaluate the prevalence and possible etiological factors associated with MIH among a group of 8–12-year-old children in Jeddah, Saudi Arabia.

## 2. Materials and Methods

### 2.1. The Sample

The present study was a cross-sectional investigation conducted at the Pediatric Dental Clinics, Faculty of Dentistry, KAU, Jeddah, Saudi Arabia, during the period of February 2011 to July 2011. All children fulfilling the following criteria were included:

1. males and females of 8–12 years of age;
2. all nationalities (Saudis and non-Saudis);
3. children who were born and were living in Jeddah;
4. children having at least one first permanent molar, erupted or partially erupted.

This study was conducted in compliance with all policies of appropriate patient care at KAU. The Ethical Committee at the Faculty of Dentistry, KAU, approved the research protocol. A written informed consent was obtained from the parents before clinical examination of their children.

A questionnaire was carefully constructed after the thorough literature review to identify all possible etiological conditions associated with MIH and related to the child or parental history, specially the mother. The questionnaire was filled out during a face-to-face interview with the accompanying parent. The questionnaire asked about the following information:

1. demographic data including the child's age, gender, nationality, place of birth, residence, as well as parents' education, and income;
2. maternal health and medications intake during pregnancy, type of delivery, possible complications during delivery or prenatal period, and child's birth weight;
3. feeding practices including type of infant feeding, duration of breast feeding if practiced, and medication use by mothers during breast feeding;
4. child's medical history during the first four years of life.

### 2.2. Clinical Diagnosis

Prior to the onset of dental examinations, one examiner was extensively trained to diagnose MIH using photographs of varying presentations of the condition. A group of 25 patients aged 8–12 years (not part of the study sample) were used for calibration (Cohen's kappa coefficient = 0.98).

**Figure 1:**
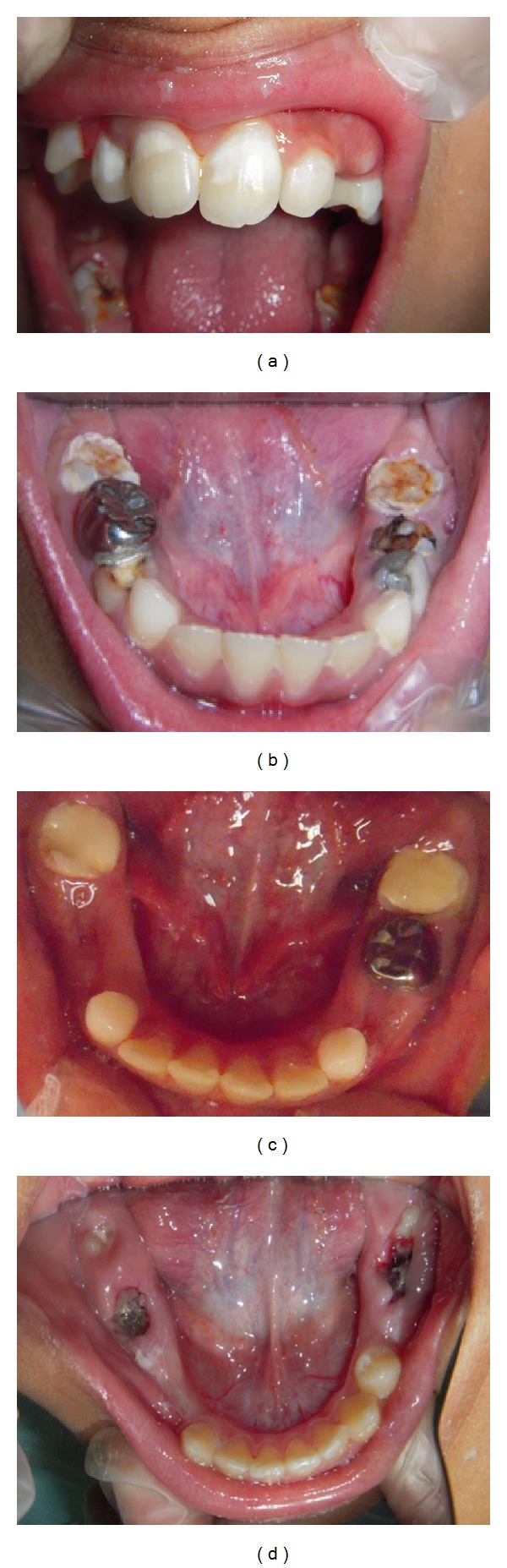
Diagnostic criteria of molar incisor hypomineralization. (a) Demarcate opacities (incisors). (b) Posteruptive breakdown (molars). (c) Atypical restorations (molars). (d) Extracted molars.

Clinical examinations of all children were performed by the trained examiner in a dental chair using mirror, dental explorer, and dental light. The criteria used for the diagnosis were based on those described in the European meeting held in Athens in 2003 [7] as shown in Table 1 and Figure 1. Prior to performing dental examinations, index teeth including incisors and FPMs were cleaned using prophylactic paste and rotary brush. A dental explorer was further used to clean the molar fissures. The examiner carefully inspected the coronal part of the FPMs and permanent incisors for demarcated opacities (distinct boundary adjacent to normal enamel), posteruptive breakdown (PEB), atypical restorations, and extracted FPMs. Children were considered to have MIH if one or more FPMs with or without the involvement of the incisors met the diagnostic criteria shown in Table 1.

The following conditions were excluded from the study:

1. dentitions with generalized opacities present on all teeth (e.g., several forms of amelogenesis imperfecta) rather than those limited to the FPMs and permanent incisors;
2. cases of fluorosis which generally tend to be diffused and generalized (affect other than target teeth);
3. the opacities occurring in permanent incisors but not in at least one FPM;
4. defects in the permanent incisors associated with history of trauma or infection in the primary dentition.

### 2.3. Statistical Analysis

The analysis of data was carried out using Statistical Package for Social Sciences Computer Software (SPSS 18.0, Inc., Chicago, USA). Descriptive statistics and chi-square tests were used to compare study variables and a probability value of less than 0.05 was regarded as statistically significant.

## 3. Results

A total of 267 children (134 males and 133 females) were included in the study. The mean age of the recruited children was 9.4 ± 1.379 years. Of these, 151 (56.6%) were Saudis (76 M, 75 F) and 116 (43.4%) were non (58 M, 58 F).

A total of 23 children were diagnosed with MIH representing an overall prevalence of 8.6%. The condition was found more among males (9.7%) than females (7.5%) and more among Saudis (9.3%) than non-Saudis (7.8%).  Table 2 shows the sample demographics and their association with the prevalence of MIH. It can be seen that none of the demographic variables was significantly related to the prevalence of MIH.

Of the 23 affected children, demarcated opacities in FPMs were the most frequent defect type (56.5%) and the only type in affected incisors (Table 3). The results show that MIH generally affected two or four FPMs (34.8%) and that the number of associated affected incisors generally declined with declining number of affected FPMs (Table 4).

**Figure 2:**
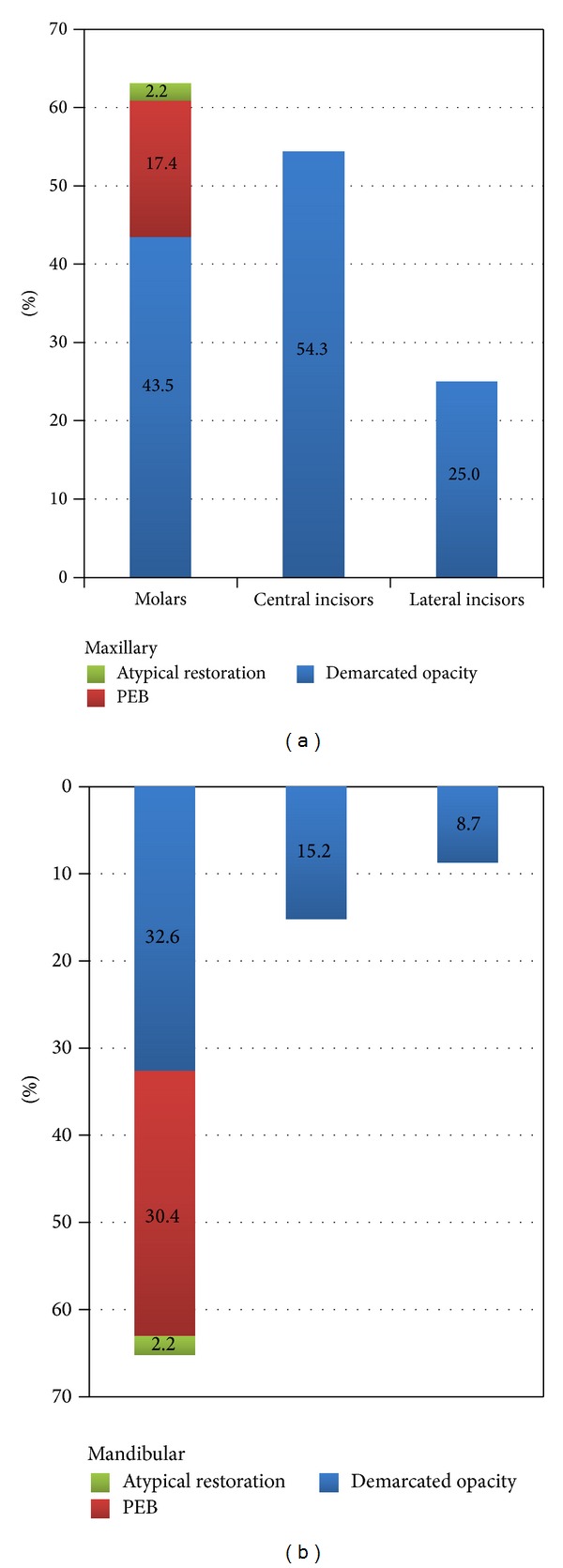
Prevalence of MIH in maxillary and mandibular permanent index teeth. The prevalence of MIH was higher in maxillary compared to mandibular central incisors (*P* = 0.01).

**Figure 3:**
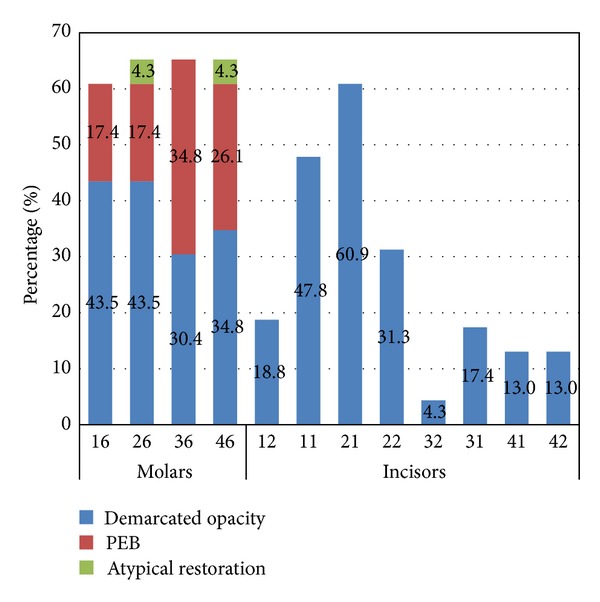
Prevalence of MIH in each permanent index tooth. Upper central incisors were more affected by MIH than lower central incisors (*P* = 0.01).

The study found that MIH had similar frequency in maxillary and mandibular molars (*X* ^2^ = 0.047, *P* = 0.5) and with nearly similar frequencies on both sides of the mouth (*X* ^2^ = 0.047, *P* = 0.5) as seen in Figure 2. The prevalence, however, was significantly higher in maxillary compared to mandibular central incisors (*X* ^2^ = 15.525, *P* = 0.01), but no difference was found between incisors on both sides of the mouth (*X* ^2^ = 0.767, *P* = 0.256). Maxillary lateral incisors were more affected than mandibular (*X* ^2^ = 3.854, *P* = 0.051), but no difference was found between both sides of the mouth (*X* ^2^ = 3.854, *P* = 0.051). The study showed that maxillary left and both mandibular FPMs were more commonly affected than maxillary right FPM (Figure 3).

The findings showed that MIH was significantly more common among children with reported health problems during the first four years of life. History of health problems was present in 82.6% of children with MIH compared to 18.4% without (*X* ^2^ = 47.486, *P* = 0.001). Children with MIH had significantly more episodes of upper respiratory tract infections including adenoiditis, tonsillitis, or asthma. They were also reported to have more attacks of fever and more antibiotics intake during these early childhood years (Table 5). On the other hand mother's illness or medication intake during pregnancy had no association with MIH, nor did the mode of delivery or complications during delivery. There was also no association between MIH and preterm birth or duration of breast feeding (Table 6).

## 4. Discussion

This study showed that MIH in Jeddah was moderately prevalent in comparison with data from other countries with approximately 2% of children having at least one first permanent molar affected. The study recruited children aged 8–12 years so that proper assessment of MIH can be done. At this age most children would have had all four FPMs and the majority of incisors, but these teeth would not have been exposed to the oral environment long enough to develop dental caries.

The finding of several affected teeth of the same tooth type may support the hypothesis of a systemic origin for MIH, which will most likely affect all teeth developing during the time period where the systemic insult had occurred. However, the study shows that not all index teeth were affected in each child and not to the same extent. It is possible that groups of ameloblasts are active at different times during the amelogenesis of individual FPMs, which might explain the asymmetry and varying severity of the defect in affected dentitions.

In agreement with previous studies, we found that demarcated opacities were the most frequent type of MIH [3, 14, 15]. The prevalence of posteruptive breakdown in our study was higher than that reported in Sweden [6], Italy [16], and Libya [17]. This may partly be explained by the inclusion of older children in our study, as some of the demarcated opacities may break down over time. This explanation is supported by findings of Wogelius et al. who reported an increased prevalence of posteruptive breakdown by increasing age [3].

The study showed that a significantly higher percentage of children with MIH had histories of illnesses during the first four years of their childhood. These findings are supported by results from other studies. A Dutch pilot study of hypomineralized first permanent molars found that respiratory diseases were common in the affected children [18]. A survey of possible etiological factors, performed on children who had one or more of their first molars extracted due to severe hypomineralization, revealed a high proportion of health problems, especially respiratory diseases [10].

Theoretically, health problems such as asthma or adenoid infections can have a disturbing effect on ameloblastic activity during enamel mineralization because of direct influence of the disease or because of hypoxia, hypocalcaemia, fever, and/or malnutrition due to the illness. Experiments have shown that conditions affecting the enamel matrix pH, that is, respiratory acidosis and abnormal oxygen levels resulting from hypoventilation in various respiratory diseases such as asthma or adenoid infections, inhibit the action of the proteolytic enzymes and the development of the crystal hydroxyapatite resulting in enamel hypomineralization [19, 20].

Corticosteroid therapy commonly used by asthmatic children is known to suppress osteoblast formation and activity, resulting in decreased bone formation [21]. A similar effect on the ameloblasts is possible and may explain how asthma can be a risk factor to MIH [22]. The association of MIH with antibiotics use is somewhat unclear. Because antibiotics are commonly used with upper respiratory infections, it is not possible to confirm whether the association was caused by the disease or the drug.

The association of MIH with fever is also inconclusive. Ameloblasts are highly susceptible to relatively minor changes in their environment. Increases in temperature [23], hypocalcaemia [24], and pH shifts [20, 25] can all disrupt the normal process of amelogenesis. Fever, however, is also a common symptom associated with most childhood respiratory infections so it may be the illness and not the fever that is causing the defect.

One possible limitation for this study may be its recall bias. Despite the extensive questioning, the obtained data may not have been a complete reflection of the child's medical history over the first four years of life. Future studies can overcome this shortcoming using the child's medical records in addition to parents' recollection. This can improve the quality of obtained medical information although it may not contain information on minor illness or treatments not advised by the physician such as the use of over-the-counter medications.

The present study shows that MIH is an existing problem in Jeddah. However, findings are not representative of the Saudi community as a whole. To better understand the condition in the country, a national oral health survey may be conducted. Because of the scarcity of data on this issue in Saudi Arabia, this study may provide baseline information which can be used for more extensive future research that can involve different regions of the country.

There is a need for longitudinal studies with large sample size to clarify the etiological role of the many possible etiological factors discussed and the value of such studies would be much increased if they attempted to determine the biological mechanisms by which these insults cause this enamel defect.

## 5. Conclusions

Findings from this study show the following.

1. The prevalence of MIH in 8–12-year-old children in Jeddah is 8.6% with no gender predilection.
2. The prevalence of MIH is significantly associated with childhood illnesses during the first 4 years of life including asthma, adenoid infections, tonsillitis, fever, and antibiotics intake.
3. There is no association between MIH and histories of birth prematurity, birth complications, low birth weight, or breast feeding duration.
4. The majority of children with MIH (65.2%) have lesions in both molars and incisors with demarcated opacities being the most frequent defect type.
5. There is no significant difference in MIH prevalence between the right and left sides of the mouth, whereas upper incisors are significantly more affected than lower ones.

## Figures and Tables

**Table 1:** Diagnostic criteria used in diagnosing MIH*.

Demarcated opacity	Posteruptive enamel breakdown (PEB)
Alterations in the translucency of the enamel, variable in degree. The defective enamel is of normal thickness with a smooth surface and can be white, yellow, or brown in color.	A defect that indicates deficiency of the surface after eruption of the tooth. Loss of initially formed surface enamel after tooth eruption. The loss is often associated with a preexisting demarcated opacity.

Atypical restoration.	Extracted molar due to MIH.

The size and shape of a restoration are not conforming to the temporary caries picture. In most cases in molars there will be restorations extended to the buccal or palatal smooth surfaces. At the border of the restorations frequently an opacity can be noticed. In incisors a buccal restoration can be noticed not related to trauma.	Absence of a first permanent molar should be compared to the other teeth of the dentition. Suspected for extraction due to MIH are opacities or atypical restorations in the other first permanent molars combined with absence of a first permanent molar. Also the absence of first permanent molars in a sound dentition in combination with demarcated opacities on the incisors is suspected for MIH. It is not likely that incisors will be extracted due to MIH.

*Based on criteria described in the European meeting held in Athens in 2003.

**Table 2:** Sample demographics.

Variables	MIH *n* (%)	Non-MIH *n* (%)	Total *n* (%)	*χ* ^2^	*P*-value
Gender					
Male	13 (9.7%)	121 (90.3%)	134 (50.2%)	0.404	0.525
Female	10 (7.5%)	123 (92.5%)	133 (49.8%)		
Age group (years)					
8	7 (7.4%)	88 (92.6%)	95 (35.6%)	2.018	0.732
9	4 (6.1%)	62 (93.9%)	66 (24.7%)		
10	4 (9.5%)	38 (90.5%)	42 (15.7%)		
11	4 (12.1%)	29 (87.9%)	33 (12.4%)		
12	4 (12.9%)	27 (87.1%)	31 (11.6%)		
Nationality					
Saudi	14 (9.3%)	137 (90.7%)	151 (56.6%)	0.191	0.662
Non-Saudi	9 (7.8%)	107 (92.2%)	116 (43.4%)		
Father's education					
Graduate education	4 (19.0%)	17 (81.1%)	21 (7.9%)	3.716	0.446
University	5 (7.0%)	66 (93.0%)	71 (26.6%)		
Diploma	6 (7.7%)	72 (92.3%)	78 (29.2%)		
Secondary	5 (7.0%)	66 (93.0%)	66 (26.6%)		
Primary	3 (11.5%)	23 (88.5%)	23 (9.7%)		
Mother's education					
Graduate education	1 (10.0%)	9 (90.0%)	10 (3.7%)	1.039	0.959
University	5 (9.3%)	49 (90.7%)	54 (20.2%)		
Diploma	6 (7.0%)	80 (93.0%)	86 (32.2%)		
Secondary	9 (9.7%)	84 (90.3%)	93 (34.8%)		
Primary	2 (10.5%)	17 (89.5%)	19 (7.1%)		
No education	0 (0.0%)	5 (100.0%)	5 (1.9%)		
Family income/Saudi Riyals/month					
Low (<5,000)	12 (8.0%)	138 (92.0%)	150 (56.2%)	1.033	0.597
Medium (5,0000–10,000)	6 (7.7%)	72 (92.3%)	78 (29.2%)		
High (>10,000)	5 (12.8%)	34 (87.2%)	39 (14.6%)		

**Table 3:** Prevalence of MIH according to diagnostic criteria.

MIH defect type	Frequency	Percent	*χ* ^2^	*P* value
Demarcated opacities	13	56.5	14.043	0.003*
Posteruptive breakdown (PEB)	6	26.1		
Demarcated Opacities and PEB	2	8.7		
PEB and atypical restorations	2	8.7		
Total	**23**	**100.0**		

*P value is significant at 0.05 level.

**Table 4:** Prevalence of MIH by type of teeth.

Number of 1st permanent molar affected	Number of children (%)	Number of children with incisors also affected (%)	*χ* ^2^	*P* value
1	5 (21.7%)	2 (40%)
2	8 (34.8%)	4 (50%)
3	2 (8.7%)	1 (50%)	6.689	0.082
4	8 (34.8%)	8 (100%)		
Total	**23 (100%)**	**15 (65.2%)**		

**Table 5:** The association between health problems during the first four years of life and prevalence of MIH.

Variables	MIH *n* (%)	Non-MIH *n* (%)	*χ* ^2^	*P* value
Adenoiditis	5 (21.7%)	6 (2.5%)	19.780	<0.001*
Fever	3 (13.0%)	7 (2.9%)	6.036	0.014*
Frequent tonsillitis	6 (26.1%)	8 (3.3%)	22.007	<0.001*
Asthma	8 (34.8%)	10 (4.1%)	31.477	<0.001*
Otitis media	1 (4.3%)	2 (0.8%)	2.355	0.125
Frequent antibiotics intake	5 (21.7%)	11 (4.5%)	11.078	0.001*
Chicken pox	2 (8.7%)	5 (2.0%)	3.637	0.057
Measles	1 (4.3%)	3 (1.2%)	1.385	0.239
GIT problems	1 (4.3%)	5 (2.0%)	0.506	0.477
Jaundice	0 (0.0%)	2 (0.8%)	0.190	0.663
Eczema	0 (0.0%)	1 (0.4%)	0.095	0.758
Urinary infections	2 (8.7%)	6 (2.5%)	2.813	0.145
No disease history	4 (17.4%)	199 (81.6%)	47.486	<0.001*
Total	** 23 (100%) **	**244 (100%)**		

*P value is significant at 0.05 level.

**Table 6:** The association between mother's medical history, delivery complications, breast feeding, and prevalence of MIH.

Variables	MIH (*n* = 23) *n* (%)	Non-MIH *n* (%)	*χ* ^2^	*P* value
Mother's illness during pregnancy	3 (13.0%)	29 (11.9%)	0.027	0.870
Mother's medication intake during pregnancy	2 (8.7%)	20 (8.2%)	0.007	0.934
Delivery mode	20 (87.0%)	212 (86.9%)	0.000	0.992
Complications during delivery	1 (4.3%)	13 (5.3%)	0.041	0.840
Preterm birth	1 (4.3%)	11 (4.5%)	0.001	0.972
Low birth weight	2 (8.7%)	23 (9.4%)	0.013	0.908
Breast feeding	21 (91.3%)	222 (91.0%)	0.003	0.959
Breast feeding duration				
<10 days	2 (9.5%)	21 (9.5%)		1.000
10 days–6 months	9 (42.9%)	98 (44.1%)	0.014	
6–12 months	7 (33.3%)	72 (32.4%)			
>12 months	3 (14.3%)	31 (14.0%)		
Mother's medication intake during breast feeding	2 (9.5%)	26 (11.7%)	0.090	0.764
Child's positive medical history	19 (82.6%)	45 (18.4%)	47.486	<0.001*

*P value is significant at 0.05 level.
